# Nurses’ Attitudes and Perceptions Towards Heart Failure Palliative Care: A Mixed Method Systematic Review

**DOI:** 10.3390/healthcare13060673

**Published:** 2025-03-19

**Authors:** Dalia Caleffi, Sara Alberti, Sergio Rovesti, Maria Chiara Bassi, Hajer Hassen, Ilaria Saguatti, Domenico Cannizzaro, Paola Ferri

**Affiliations:** 1 Clinical and Experimental Medicine PhD Program, Department of Biomedical, Metabolic and Neural Sciences, University of Modena and Reggio Emilia, 41121 Modena, Italy; 2 Department of Biomedical, Metabolic and Neural Sciences, University of Modena and Reggio Emilia, 41121 Modena, Italy; 3 Azienda Unità Sanitaria Locale di Reggio Emilia, 42122 Reggio Emilia, Italy; 4 Maternal and Child Department, Neonatology Operating Unit, Azienda Ospedaliera Universitaria di Modena, 41125 Modena, Italy

**Keywords:** heart failure, palliative care, nursing, attitude, perception

## Abstract

**Background/Objectives:** Heart failure assistance is strictly correlated with the concept of palliative care. Supportive treatment should be part of the heart failure patient pathway from the beginning. Palliative care with interprofessional effective collaboration could be an important resource used to reduce heart failure distressing symptoms and improve quality of life. Nurses, as professionals with a holistic vision of care, play a crucial role in palliative care introduction and implementation. The aim was to explore nurses’ attitudes and perceptions of heart failure palliative care, updating and adding knowledge to the current evidence. **Methods:** A systematic mixed-method review following the Joanna Briggs Institute methodology was undertaken. The screening of articles, data extraction and quality appraisal were performed by more than one author. The search was undertaken in May 2024 and applied to PubMed, Cinahl, Embase, Web of science, PsycInfo, Cochrane library and Scopus. A convergent integrated approach allowed us to combine qualitative and quantitative data. The analysis and synthesis of results was guided by the Theoretical Domain Framework. **Results:** Of the 1048 records identified, 26 met the inclusion criteria. Twelve framework domains were completed with data extracted. A flow chart was elaborated to offer an overview of the main concepts included. **Conclusions:** Numerous behaviors and elements influenced heart failure palliative care implementations. Analysis has shown that each analyzed element was strictly correlated one with another. When implementation was possible, outcome improvement sustained palliative care benefits with the direct involvement of nurses as educators and coordinators.

## 1. Introduction

Palliative care is a comprehensive and supportive care program that is recommended for all people with distressing symptoms [1]. The belief that palliative care is delivered solely to patients who are about to die [2], and destined exclusively for patients dying or suffering from cancer, exists [3].

The prevalence of people living with chronic non-communicable diseases is increasing dramatically. There are numerous chronic diseases by which adults may be affected, and the correlated symptom burdens are very important [4].

The World Health Organization (WHO) underscores that 56.8 million people need palliative care, but less than 50% of people with non-communicable diseases are reached by their services [5].

Heart failure is a clinical syndrome consisting of cardinal symptoms such as breathlessness, ankle swelling and fatigue; it is accompanied by signs (e.g., elevated jugular venous pressure, pulmonary crackles, and peripheral edema) and correlated with elevated intracardiac pressures and/or inadequate cardiac output [6].

The Heart Failure Society of America 2024 states that the lifetime risk of heart failure has increased to 24%, and the prevalence is expected to rise to 8.7 million in 2030, 10.3 million in 2040, and 11.4 million by 2050 [7]. The European Society of Cardiology (ESC) 2021 reported a prevalence of 1–2% in adults. This prevalence is probably higher since studies reported only recognized/diagnosed heart failure cases.

Patients with heart failure have the same distressing symptoms and correlated symptom burdens as those with cancers. In the most advanced stages, the number of symptoms exceeds those associated with advanced cancer [3,8]. The disease trajectory of each heart failure patient is unique but characterized by a gradual decline, which leads to team-based approach palliative care evaluations [6].

As studies have evidenced, heart failure palliative care is correlated with multiple difficulties due to the unpredictable course of the disease, the lack of specific education and communication, and misconceptions [3,9].

The literature in primary and secondary studies on heart failure palliative care is increasing.

Different types of reviews—narrative, systematic and integrative review—have been conducted. However, they explore different palliative care aspects such as cost effectiveness [10], tools [11], carer needs [12], interventions [9], and nurse opportunities to overcome barriers [13], with different methodological aspects.

Bagheri et al. (2022) [1] analyzed palliative care heart failure guidelines’ quality, and they evidence a low score of these guidelines’ applicability due to a lack of discussion about facilitators, barriers, potential sources of recommendations, and monitoring and/or audit criteria.

The WHO recognizes the importance of nurses as part of the multidisciplinary teams involved in palliative care [5].

Nurses play a crucial role in palliative care delivery. Nurses often recognize changes in patient health status, providing an understanding of the right moment to consider palliative care [14]. Indeed, they are holistic providers whose role is crucial in delivering palliative care, which is also influenced by their attitudes [15,16]. Research has demonstrated a positive impact of palliative care led by nurse involvement when barriers are overcome [14]. Across palliative care settings, nurses have a unique position, with a broad range of abilities and activities [17].

As part of the interprofessional approach on which palliative care is based [1], understanding the point of view of each specific team member may help improve palliative care’s effective implementation with correlated benefits for the patients and carers involved.

The purpose of this systematic review was to elaborate an overview of nurses’ attitudes, experiences and perceptions about heart failure palliative care using a methodology that allows integrated results derived from qualitative and quantitative studies. Deepening knowledge of these topics may help to identify facilitators and barriers to effective palliative care implementation. Clear identification may allow interventions and improvement in clinical practice. The published articles mostly analyze heart failure palliative care without focusing merely on the nurses’ point of view.

### Aims

The primary aim of this systematic mixed method review is to explore nurses’ attitudes and perceptions regarding heart failure palliative care.

The main questions that the systematic revision intends to answer are as follows:

1—What are nurses’ attitudes and experiences of heart failure palliative care implementation?

2—What are nurses’ perceptions of effective heart failure palliative care applicability?

The secondary aim was to distinguish nursing interventions according to the caring phase—onset of heart failure, decompensation, end of life care and advanced care planning.

## 2. Materials and Methods

### 2.1. Design

A systematic mixed method review was conducted following the Joanna Briggs Institute (JBI) guidelines [18]. This method was chosen to evidence all the potential eligible information about nursing attitudes, experiences and perceptions. A first review of the literature, in fact, showed that the potentially pertinent studies conducted differ in methodological aspects.

This systematic mixed method review is reported using the preferred reporting items for systematic review and meta-analysis (PRISMA) statement criteria [19].

The review protocol has been registered in Prospero https://www.crd.york.ac.uk/prospero/display_record.php?ID=CRD42024573570 (accessed on 29 December 2024).

### 2.2. Search Method

The search strategies were developed with the support and the guidance of an expert informatician and academic librarian. In order to find all the available evidence, a specific research strategy was adapted for each interrogated database, using keywords as indexed terms. It was applied to many sources—Cinahl, Psycinfo, Embase, Scopus, Cochrane Library, PubMed and Web of Science. Every article, either in English or in Italian, corresponding to inclusion criteria was selected. The search was undertaken in May 2024 with no limitation applied to publication date.

After performing the searches (Table 1), duplicate records were eliminated and screened by title and abstract by two independent reviewers. Any disagreement was actively discussed with the collaboration of a third researcher in case of discordance. To minimize the risk of bias, the Rayyan software (https://help.rayyan.ai/hc/en-us/articles/4406419348369-What-is-the-version-of-Rayyan, accessed on 15 February 2025) was used to ensure the blindness of the process [20,21]. With the same procedure, the full texts of the potentially eligible studies were analyzed. Reference lists of screened eligible articles were reviewed to find other eligible articles.

Studies were considered for inclusion when the eligibility criteria were met. The inclusion criteria were applied to all studies in which the utilized method was clearly defined, and the full text of the article was available. Primary qualitative, quantitative and mixed-methods studies were included. Mixed-method studies were only considered if data from quantitative or qualitative components could be clearly extracted.

Secondary literature studies (e.g., reviews, meta-analyses, study synopsis), descriptive articles that did not report data collected on a sample of subjects, case reports, case series, and expert opinions were not included. Articles of secondary literature studies were evaluated for inclusion, while review articles were excluded. Our aim was in fact to extract data from primary studies and to elaborate a mixed method systematic review.

Studies involving children or patients aged less than 18 were excluded, given the particular needs of this segment of population.

The PICO of the included studies summarizes the two aim questions described above. The acronym includes “P” as heart failure patients for whom nurses’ palliative aspects of care were implemented or considered; “I” is nurses’ attitudes and perceptions of palliative care implementation; “C” is usual practice and any kind of different intervention, if present; “O” is outcomes related to nursing palliative care attitudes, perceptions, and interventions in palliative heart failure care.

The expression “nurses’ attitudes and perceptions” refers to all the aspects of evidence-based practice implementation considered as triggering issues or opportunities.

The main outcomes that the systematic mixed method review intended to study in depth were nursing palliative care aspects for heart failure patients.

Other outcomes that the systematic review aimed to explore were the specific interventions with nursing involvement in heart failure palliative care.

The critical assessment of included studies was conducted by two researchers using the JBI Critical Appraisal Tools—Checklist for Randomized Controlled Trials [22], Checklist for quasi-experimental study [23], Checklist for Prevalence study [24], Checklist for analytical cross-sectional study [25], Checklist for cohort studies [25] and Checklist for qualitative studies [26]. Quantitative and qualitative aspects of mixed-method studies were evaluated using the corresponding JBI checklist. After independent evaluations, any disagreement was discussed by the two members of the team. In case of discordance, a third one was involved. Quality appraisal was evidenced and considered in the interpretation and discussion of results.

Data of eligible studies were extracted by first and second authors following the Joanna Briggs Institute indications. Aspects included in the data extraction format were the type of study, country, setting, aim, characteristics of participants, age and sex of participants, training, heart failure population type, type of palliative care, outcomes defined, data collection, period of data collection, data analysis, key findings and limitations.

To have a greater depth of understanding of the phenomena, the convergent integrated approach of the JBI mixed-methods systematic review guidelines was implemented. Data were synthesized by two researchers with continuous comparison and discussion. Convergent synthesis refers to the process of combining data extracted from quantitative research and qualitative research. Quantitative data were “qualitized” as textual descriptions through narrative interpretation. Qualitative data were firstly extracted, identifying findings, categories and synthetized findings.

Hence, nursing attitudes and perceptions towards palliative care practice were categorized using the Theoretical Domains Framework (TDF) revised by Cane et al. (2012) [27]. TDF identifies 14 domains in which categorized behavior influences the implementation of the evidence base.

Summarizing nursing attitudes and perceptions in the specific setting of heart failure palliative care enabled us to identify all variables that have to be considered to undertake palliative care in heart failure patients.

In order to synthesize the existing knowledge, tables have been used to present the key concepts identified through the analysis in which the synthetized findings were reported.

## 3. Results

### 3.1. Selection of Sources

**Figure 1:**
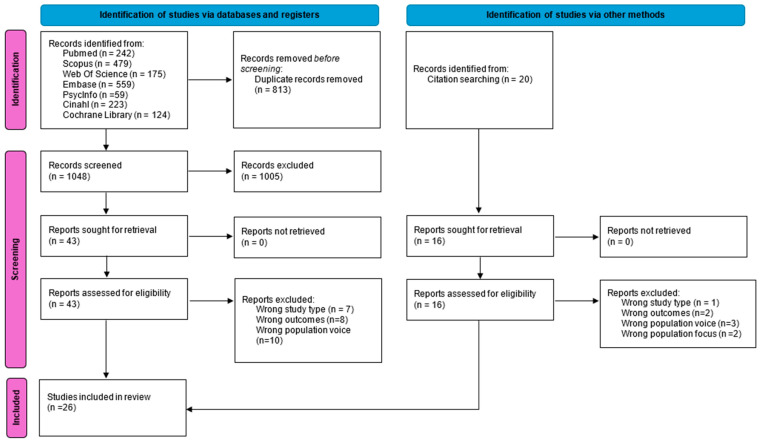
Flow diagram for selection of studies (PRISMA FLOW DIAGRAM).

Database searching identified 1861 articles; after removing duplicates, 1048 articles were screened for title and abstract. Among these, 43 articles were included for full-text review. Following a full text review, 18 articles were included. Another 16 articles that were potentially eligible were identified from full-text reference analyses, from which 8 were excluded. A total of 26 articles are included in this review. An outline of the study screening and selection process is presented in the PRISMA flowchart shown in Figure 1 [19].

### 3.2. Critical Appraisal of Sources of Evidence

Seven randomized controlled trials were appraised using the JBI Critical Appraisal Tools: Checklist for Randomized Controlled Trials. For all studies, patient and assessor blindness was not applied [28,29,30,31,32,33,34]. Therefore, four assessors were not blind to treatment assignment, or the report was unclear about the item [28,30,33,34]. Thirteen studies were evaluated through the qualitative checklist [35,36,37,38,39,40,41,42,43,44,45,46,47]. Most of them demonstrated a lack of information or an absence of researcher influence on the study evaluation.

The critical aspect of the cohort study analyzed was the absence of confounding factors considered [48]. A cross-sectional study was conducted for almost all checklist items [43]. Patient characteristics were not described in detail by all [43,49,50,51], and three [49,50,51] of them did not clearly describe inclusion criteria.

Prevalence studies demonstrated some critical aspects related to the detail of the sample and coverage and response rates [42,52].

No study was excluded based on quality evaluation. Critical appraisal was fundamental to the discussion and interpretation of the results, and to deriving an understanding of the studies’ characteristics.

### 3.3. Characteristics of Source of Evidence

Table 2 outlines the included studies. Most employed a qualitative design (n = 11) [35,36,37,38,39,40,41,44,45,46,47]. Ten employed the interview format for data collection. Four studies were conducted in the United Kingdom [38,39,41,45], two in Iran [35,40], three in Australia [36,44,46], one in Sweden [37] and one in Germany [47]. Quantitative studies were mostly randomized controlled studies (n = 7) [28,29,30,31,32,33,34], with one pilot pre- and post-test study [53], one pragmatic nonrandomized study [51], one descriptive study [48] with a cohort method, and three cross-sectional studies [43,49,50]. Two mixed-method studies [42,52] with qualitative data and a survey were included.

Qualitative interviews or questionnaire were directed only towards nurses in four studies [35,36,37,42]. Other samples consisted of an interprofessional team, with carer involvement in two cases [38,40]. Randomized controlled studies evaluated the efficacy of the intervention analyzed for samples of patients [28,29,30,31,32,33,34]. The observational cohort and cross-sectional studies observed nurses working in heart failure palliative care [43,48,49,50]. The pilot quasi experimental study focused on nurses’ outcomes improvement after educational intervention [53]. The survey was submitted to nurses, physicians and other healthcare professionals. Mixed studies evaluated only nurses’ positions.

### 3.4. Nurses’ Attitudes and Perceptions

**Figure 2:**
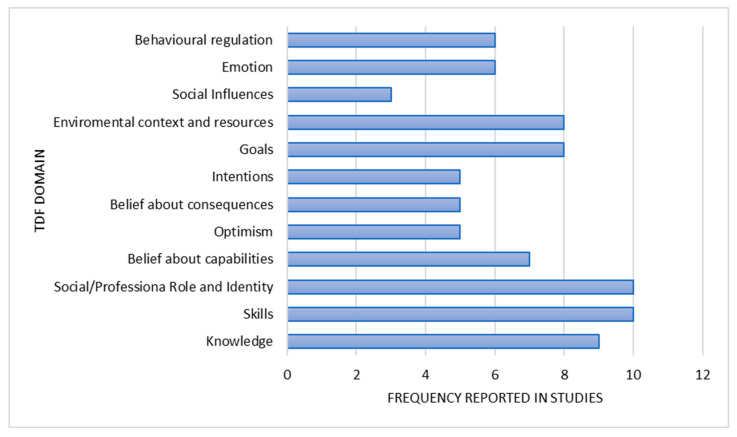
TDF domains frequency.

Qualitative and qualitative data about nurses’ attitudes and perceptions were allocated to twelve of the fourteen TDF domains (Figure 2, Table 3).

#### 3.4.1. Knowledge

Knowledge was a theme discussed by nurses. Patients and carers were described to have unrealistic expectations regarding diagnosis and trajectory. Education and information were not always given by doctors when it was perceived that patients had to have all treatment options. Professionals and nurses knew that each patient was unique and that knowledge improved attitudes. Knowledge about palliative care and chronic illness is important. Clinicians were lacking in palliative care for heart failure management and did not know how to delay all symptoms. Knowledge also implies knowing who is responsible for providing palliative care. Nurses knew and understood the concept of working in a team.

#### 3.4.2. Skills

Recognition of when to start palliative care was considered difficult, and it often happened too late. Relapses offer indicators for considering palliative care. Nurses were better than clinicians at talking about palliative care and at considering constructing a good relationship and related communication with the patient from the beginning. Nurse specialists who could provide end-of-life palliative care were lacking due to a lack of master’s degrees in the sector.

#### 3.4.3. Social/Professional Role and Identity

Palliative care team involvement and consultation was desirable. Interprofessional involvement, cooperation and communication are needed. It could be painful or helpful, but it is important. Multiple ideas and poor communication could become an obstacle to palliative care implementation. Defining roles and duties was perceived as fundamental. Communicating, asking for advice and reflecting on difficult situations were outlined elements, and discussion across team members helped the learning process.

#### 3.4.4. Beliefs About Capabilities

Communication abilities between members of an interdisciplinary team were described as fundamental to help patients achieve good death. Sometimes, communication abilities were also reported as weak and fragmented. Nurses understood the importance of discussing prognosis, but they hesitated if clinicians had not already done so. They did not feel comfortable when perceiving a lack of education in discussing prognosis, which was considered a physician’s responsibility.

#### 3.4.5. Optimism

Patients’ false hopes were described. They were optimistic when included on the transplant list because they did not have cancer, without recognizing the chronic life-limiting condition. Patients’ and families’ thoughts were unrealistic and they did not accept limited further interventions. Younger nurses did not always understand mortality. Professionals perceived patients’ limited awareness of the gravity of the situation.

#### 3.4.6. Beliefs About Consequences

Patients and families identified palliative care as giving up. Nurses were afraid to frighten people or take away their hope by talking about the end of life. Specific palliative care education was considered beneficial in allowing the integration of palliative care and relative signs’ recognition.

#### 3.4.7. Intentions

Prognosis was a difficult topic to address in heart failure assistance. It is described as important. Clinicians desired to talk about prognosis, but it was not always possible. Some people appreciated discussing the prognosis others did not want.

Transition to palliative care was considered by physicians as a failure, and team spirit was lacking.

#### 3.4.8. Goals

Nurses described heart failure as an illness with an unpredictable trajectory correlated with a difficulty in understanding when to start palliative care. It was difficult to understand when to begin focusing on symptoms or medical treatment. Patients were not aware of the chronic long-term progressive illness. Death acceptance and discussions about death were important. Professionals would have preferred to be honest with patients when nurses and clinicians perceived the need to initiate palliative care and correlated goals in different moments. Cardiologists fixated on heart failure mechanisms without thinking about well-being or death.

#### 3.4.9. Environmental Context and Resources

Limited resources, financial constraints, and little time to build relationships were some of the obstacles described by nurses. Professional education was a huge need when there was a shortage of specialist medical professionals and services in the field of palliative care. The homecare setting was considered easier for people to be themselves.

#### 3.4.10. Social Influences

Health system support in providing palliative care services was not always perceived, and societies might not want to introduce, speak about or plan for death. In some contexts, no-resuscitation laws were present. A gap in identifying who is responsible to provide palliative care is sometimes described.

#### 3.4.11. Emotions

Nurses felt nervous, constantly anxious, and tired when taking care of end-of-life heart failure patients, drowning in their suffering. They felt angry and frustrated about resuscitating people when they identified it as not appropriate. Frustration emerged when nobody made decisions about palliative care, or when it was difficult to talk about death in a homecare setting. Positive feelings arose instead when patients relied on them in communicating their feelings. The family and patients did not always desire to talk about death. Some welcomed it, while others denied that patients’ conditions were getting worse.

#### 3.4.12. Behavioral Regulation

Nurse involvement as case managers in disease and symptoms management has a positive impact on multiple dimensions: severity of symptoms, physical and mental functioning, satisfaction, admission, and quality of life. The leadership or nurse coordination of a team is correlated with positive outcomes. Psychoeducational interventions and virtual programs are some of the methods used to improve patient outcomes.

### 3.5. Nurses’ Interventions

Quantitative studies’ interventions and their correlated outcomes are synthesized in Table 4. Ten different types of interventions were identified. None of the studies evidenced a negative impact of the intervention analyzed. Regarding outcomes explored, only in a limited number of situations was there no difference in the intervention implementation found [28,31].

## 4. Discussion

This systematic review evaluates nurses’ attitudes and perceptions about heart failure palliative care. A mixed-method methodology allowed us to integrate experiences and effectiveness so as to derive a deeper understanding of the topic [18].

We classified nursing attitudes, experiences and perceptions using TDF domains developed by Cane et al. (2012) [27]. This framework was developed to explain implementation problems, inform implementation interventions, and bring about behavioral change. It could be applied to either qualitative or quantitative data [27]. A summary of the findings is presented with Table 5. Knowledge was a theme that affected all care participants. Nurses highlighted that patients and carers have unrealistic expectations regarding diagnosis and trajectory. A lack of information regarding heart failure severity and prognosis is an important barrier to palliative care implementation, described also by Schallmo et al. (2019) [54]. Clinicians and nurses were aware of the importance of knowledge to improving attitudes. However, information was not always given, and clinicians were lacking in training about symptom management. Nevertheless, symptom burden associated with heart failure is comparable to, or exceeds, that of cancer patients [9].

Knowledge should be improved considering not only health professionals’ training and educations, but also enhancing patient’s awareness and disease knowledge. Although cardiologists should be able to assess and treat heart failure symptoms, it is also important to consider specific palliative care specialists’ involvement [55]. Poorly delivered information is strictly correlated with communication. Communication and education about treatment options are fundamental to increase healthcare professionals’ knowledge, attitudes and preparedness to practice [54]. A lack of knowledge about who was responsible for providing palliative care emerged. Oishi et al. (2014) [56] evidenced this problem in 2014, describing how each professional involved expects other specialists to take more responsibility in patients’ palliative care. They underlined the importance of coordinated care, recognizing the presence of many barriers correlated with a lack of resources and time to maintain the shared vision, mutual respect and inclusive decision-making. These are the same barriers that were identified by our systematic review. Responsibility also emerged through the prognosis concept. Nurses described the topic as difficult. It is not clear who is responsible for talking about prognosis and patients do not always appreciate talking about it. Prognosis represents an obstacle related to the concept of intentions. Prognosis is in fact correlated with the concept of shared decision-making. Timing and communication in relation to prognosis are also difficult in the case of cancer patients [57]. In noncommunicable non-cancer diseases, such as heart failure, understanding prognosis is more difficult because of the unpredictable nature of the illness characterized by episodic, acute exacerbations, frequent hospitalization, stabilization and steady decline. Predicting and talking about prognosis is, however, important to estimate healthcare utilization and to identify groups that may benefit from specific intervention [58,59]. However, tools and scores available to estimate prognosis were prevalent in relation to cancer disease [58]. There are wide variations in access to palliative care in people with heart failure compared to those with cancer [59]. We believe that it is possible to state that communication should not be overlooked in clinical practice. It is in fact an important theme strictly correlated with healthcare professionals’ effective communication within their team, but also with patients and families. If adequately implemented, it could contribute many barrier reductions.

Moreover, our results show that patients were not always aware about the illness’s gravity, and did not recognize the chronic life-limiting conditions. As the study of Shropshire et al. (2023) [60] shows, heart failure awareness is still lacking, and it is influenced by multiple factors, such as numbers of opportunities to meet healthcare providers, the lack of symptoms or nonspecific symptoms, and an identifiable heart failure cause as the consequence of myocardial infarction. Lack of awareness is a problem that starts in the general population, who have insufficient knowledge of the symptoms, causes and treatment methods of this disease [61]. Awareness, poor understanding of the disease and a lack of belief in treatment are some of the factors influencing a patient’s adherence to treatment [62].

Starting palliative care for heart failure patients and carers was perceived as giving up. Consequently, nurses were afraid to talk about prognosis, end of life and death. Understanding when to start palliative care or focusing on symptoms rather than medical treatment is limited by the unpredictable illness trajectory and patient awareness of illness. The literature contains evidence that it should be necessary to consider palliative care as part of the overall treatment due to the unpredictable course of the disease [3]. Clinicians sometimes are more able to predict death or end-of-life care due to the severe and refractory symptoms that are present [2]. Tools and scores are prevalent in relation to cancer disease, and now some have also been developed for heart failure patients, such as the “needs assessment tools progressive disease: heart failure” (NAT: PD-HF) [11,58,63].

Nurses identified the importance of education for the implementation and integration of palliative care services. Education is a huge need. The literature has already evidenced that structured nursing student education may improve and encourage attitudes regarding providing care to patient who are dying [64]. However, limited resources and financial constraints have been described as obstacles to palliative care. The lack of specialists and services is a problem that has already been described. The WHO, in an article published in 2021, underlined that the provision of palliative care in most countries lags far behind the need for these essential services. Further elements on which to focus attention include the fact that health system support in providing palliative care was not always perceived, and societies might not want to introduce, speak about or plan for death, or set specific laws on some aspects of palliative care. Healthcare systems should take into consideration the importance of palliative care, differentiating intervention based also on cultural beliefs. As such, it may be possible to avoid unnecessary expenses or later interventions. The lack of palliative care treatments is a problem described across the world, in which cultural aspects should be considered, particularly in multicultural societies in which the dominant culture affects life opportunities at both group and individual levels in lower social strata [65]. In many countries, there is a progressive and growing acceptance of legal support as a key component of holistic palliative care to defend and respect human rights. Ezer et al. (2018) [66] and Osei et al. (2022) [67] described the progress made by countries such as Romania, Hungary, Georgia, Malawi, Kenya and East Asian countries. As these studies have demonstrated, legislation about palliative care is increasing, but differences already exist across the countries, and many developments still have to be encouraged.

A theme that permeates, and which is the consequence of all the topics analyzed, regards the emotions felt by nurses, patients and carers. Nurses described themselves as anxious, tired, frustrated and angry due to the patients’ suffering, and thus encountered difficulty in palliative care implementation.

Experimental studies demonstrated positive results, supporting the importance of building good-quality palliative care programs.

Interdisciplinary collaboration was mostly implemented [28,30,33,34,51]. People receiving the palliative program experienced positive effects on symptoms frequency and severity, health status, and quality of life. Clear evidence of hospital admission reductions did not emerge, possibly related to the type of intervention delivered.

These interventions were focused on the introduction of a nurse with palliative competence who evaluated patients’ symptoms and supported them throughout educational interventions or the coordinated interventions of the interdisciplinary team. The activities aligned with the needs that emerged from the qualitative aspects.

**Figure 3:**
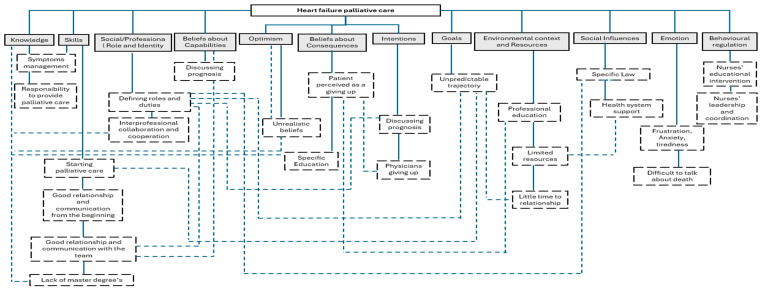
Summary flowchart of main topics. Legend: solid lines TDF identified, dashed lines findings and correlations between TDF domains.

Finally, due to the importance of palliative care intervention implementation to reduce barriers, avoid negative feelings and foster good practice, we have elaborated a flowchart based on the results (Figure 3). This flowchart attempts to summarize TDF elements, showing strict internal correlations.

The flowchart helps us to understand that acting on an element may have a positive impact on the correlated ones. Some main themes and recurrent issues emerged from our results. Knowledge, education and communication concern all TDF domains addressed by nurses’ attitudes and perceptions. They may be considered associated with the capability, opportunity and motivation components of the target ideal behavior [27]. The TDF framework enables us to have a clear view of nurses’ attitudes and perceptions, allowing us to categorize and closely analyze the results. Behavioral change in clinical practice should, in fact, begin with an analysis of which elements could influence them to explain implementation problems and construct implementation interventions. Improvements in knowledge, communication and education could have a positive impact on other potential barriers, such as the unpredictable course of the disease and differences in patients’ symptom burdens in progressive heart failure stages.

As described by WHO (2021) [68], palliative care is a human right and a moral imperative that should not be neglected. Implementation helps improve the quality of life of people who are dealing with the challenges posed by life-threatening illnesses.

### Strenghs and Limitations

It is fundamental to underline that it was not possible in our analysis to distinguish interventions and attitudes according to the heart failure stage, since the studies did not clearly specify it. Transplant and left ventricular assistance device (LVAD) patients were not considered due to the specific interventions needed. Other specific reviews may be useful in enriching the results obtained.

We acknowledge heterogeneity in study design and intervention type. Consequently, we adopted a convergent-integrated design. This method allows us to generate a deeper understanding of the topic from multiple points of view. Regarding this aspect, it is important to think about it as a limitation, but also as a strength. Nevertheless, it is important to be careful in forming the results and generalizations due to the different study methodologies and quality appraisal levels.

Language restrictions (English or Italian) should also be considered, as the vast majority of articles are now published in the English language.

## 5. Conclusions

Nurses’ attitudes and perceptions towards heart failure palliative care are characterized by many influences and behaviors that are strictly correlated with one another. Communication, knowledge and education emerged as important topics that influence palliative care implemantation. Palliative care among the heart failure population is recognized as important, but misconceptions and a lack of knowledge primarily on the part of patients and clinicians may exist. As such, the risk of hindering the real impact of palliative care is present. Evidence has indicated that nurses’ contributions, with educational interventions and leadership, improve patients’ outcomes and palliative care implementation. As researchers and practitioners, we must consider the complexity of such types of care, illnesses and elements involved. Understanding the correlation between factors that can influence palliative care implementation, as we have tried to underline, may help us to understand the starting point for improving heart failure palliative care implementation.

## Figures and Tables

**Table 1:** Search strategy, focus of interest and exclusion motivations.

Database	Search Strategy	Focus of Interest
Cinahl	((MH “Palliative Care”) OR TI (palliative care OR palliative treatment* OR palliative therap*) OR AB (palliative care OR palliative treatment* OR palliative therap*)) AND ((MH “Heart Failure”) OR TI (heart failure OR cardiac failure) OR AB (heart failure OR cardiac failure)) AND ((MH “Nurses”) OR TI nurs* OR AB nurs*)	To explore nurse attitudes and perceptions of heart failure palliative care
		To update the current literature focusing primarily on nurse involvement
Psycinfo	(exp Palliative Care/OR (palliative care or palliative treatment* or palliative therap*).ab,ot.) AND ((heart failure orcardiac failure).ab,ot.) AND (exp Nurses/OR “nurs*”.ab,ot.)	**What is expected to understand**
		Nurses’ attitudes and experiences of heart failure palliative care Barriers and facilitators correlated with heart failure palliative care, implementation
Embase	(‘palliative care’:ab,ti OR ‘palliative treatment*’:ab,ti OR ‘palliative therap*’:ab,ti OR ‘palliative therapy’/mj) AND (‘heart failure’:ab,ti OR ‘cardiac failure’:ab,ti OR ‘heart failure’/mj) AND (nurs*:ab,ti OR ‘nurse’/mj) AND ([english]/lim OR [italian]/lim)	
Web of Science	(palliative care OR palliative treatment* OR palliative therap* (Title) or palliative care OR palliative treatment* OR palliative therap* (Abstract)) AND (heart failure OR cardiac failure (Title) or heart failure OR cardiac failure (Abstract)) AND (nurs* (Title) or nurs* (Abstract)) and English (Languages)	
		**Exclusion criteria**
Cochrane Library	(MeSH descriptor: [Palliative Care] explode all trees OR (palliative care OR palliative treatment* OR palliative therap*):ti,ab,kw) AND (MeSH descriptor: [Heart Failure] explode all trees OR (heart failure OR cardiac failure):ti,ab,kw) AND (MeSH descriptor: [Nurses] explode all trees OR (nurs*):ti,ab,kw)	Study methodology not clearly explained Secondary literature studies Nurse voices not differentiable from other participants potentially involved Involvement of only other healthcare professionals Heart failure care without considering palliative care aspects or pathway Role of professionals involved not differentiable
Scopus	(TITLE-ABS-KEY (palliative AND care OR palliative AND treatment* OR palliative AND therap*) AND TITLE-ABS- KEY (heart AND failure OR cardiac AND failure) AND TITLE-ABS-KEY (nurs*)) AND (LIMIT-TO (DOCTYPE, “ar”) OR LIMIT-TO (DOCTYPE, “re”)) AND (LIMIT-TO (LANGUAGE, “English”))	
PubMed	(“Palliative Care”[Mesh]) OR Palliative care[Title/Abstract] OR Palliative Treatment*[Title/Abstract] OR Palliative Therap*[Title/Abstract]) AND (“Heart Failure”[Mesh] OR Heart failure[Title/Abstract] OR Cardiac Failure[Title/Abstract]) AND (“Nurses”[Mesh] OR nurs*[Title/Abstract])	

Legend: * plural or singolar words.

**Table 2:** Characteristics and main results of the studies considered in this review.

Study, Location	Design	Participants, Type of Palliative Care	Summary Quality Score	Aim	Key Findings
Akbarian-Rokni et al., 2023 [35] Iran	Qualitative study	33 nurses of two cardiac referral educational centres End of life heart failure palliative care	90%	To explore Iranian nurses’ perceptions of the challenges involved in providing end-of-life care to people with heart failure.	In the Iranian context nurses have difficulty in applying palliative care because of the lack of service, adequate training, lack of single instructions in the country, poor support and team communication. Nurses often experience fatigue and emotional conflict.
Borbasi et al., 2005 [36] Australia	Descriptive exploratory qualitative study	17 nurses (12 registered nurses across acute care sites, 5 community nurses involved in palliation at home or hospice), end stage heart failure palliative care	80%	To generate a rich description of nurses’ experiences of caring for patients who are dying from heart failure; to generate new understandings and insights from this information and make it available to nurses and other healthcare workers.	It is important to offer a patient the opportunity to die at home with palliative care support. At the hospital, the provision of quality care at the end of life continued to represent a significant organizational, cultural and political challenge. Structural and organizational changes would be essential to provide the necessary skills, time, resources, collaboration and support for effective palliative care.
Brannstrom et al., 2005 [37] Sweden	Qualitative study with phenomenological-hermeneutic method	11 homecare nurses, advanced congestive heart failure palliative care (NYHA III–IV)	90%	To illuminate the meaning of being a palliative nurse for persons with severe congestive heart failure in advanced homecare.	Participating in the patient’s everyday life means being a guest in the patient’s home; being trustworthy, preserving calm and security; and being adaptable to the patient’s way of life.
Browne et al., 2014 [38] United Kingdom	Qualitative study	30 advanced heart failure patients, 20 carers and 65 professionals (including district nurse, heart failure nurse, palliative nurse, Marie Curie nurse) advanced heart failure palliative care	90%	To understand challenges and identify what needs to be done to improve palliative care comparing the perspectives of patients, caregivers, and professionals.	Uncertainty about implications of diagnosis, treatment in patients with heart failure; healthcare services that are poorly coordinated and offer fragmented care; health professionals with a lack of knowledge, opportunities, or adequate support; the need to improve care coordination and communication between patients, their families, and healthcare professionals.
Fairlamb and Murtagh., 2021 [39] United Kingdom	Qualitative interview study	16 health professionals including 1 specialist heart failure nurse, 2 specialist respiratory nurses, and 4 specialist palliative care nurses. End-stage palliative care heart failure	80%	To explore health professional perceptions and current practices in relation to specialist palliative care for patients with end-stage cardiac disease.	It is difficult to understand when to start palliative care, given the lack of advance care planning discussions, poor communication, lack of health professional time and confidence, lack of knowledge and limited specialist palliative care involvement in multidisciplinary teams.
Glogowska et al., 2016 [41] United Kingdom	Qualitative in-depth interview study	24 healthcare professionals (including specialist heart failure nurses in secondary care and community care). Advanced heart failure palliative care	80%	To explore the perceptions and experiences of healthcare professionals working with patients with heart failure around end-of-life care.	Lack of communication between health professional, patients and families; importance of discussing with patients about death and dying; difficulty of recognizing when heart failure patients are approaching the terminal phase of their condition; to find alternatives to hospital admission for patients in end-of-life palliative care.
Motlag et al., 2023 [40] Iran	Qualitative study	15 participants, including 6 patients, 2 family caregivers, and 7 healthcare team members (4 nurses, a psychiatric nurse, a nutritionist, and a PC physician). Heart failure palliative care	80%	To explain the barriers and facilitators of palliative care in older adults with heart failure.	The findings of this study explain the barriers and facilitators of palliative care in older adults with heart failure. Removing the barriers and supporting the facilitators gives older adults with heart failure better access to palliative care. Therefore, to expand palliative care centers for older adults with heart failure, health system officials and policymakers should pay attention to organizational infrastructures and remove the barriers at organizational, social, educational, and economic levels with the cooperation of governmental organizations, benefactors, and nongovernmental organizations.
Singh et al., 2020 [44] Australia	Qualitative study	15 participants (cardiologists, palliative care specialists, heart failure nurses and palliative care nurses) of which 5 were heart failure nurses and 1 was a palliative care nurse. Heart failure palliative care	80%	To explore healthcare professionals’ perspectives on access to palliative care for patients with chronic heart failure, focussing on patient, provider and system factors.	Patients’ themes that emerged were patient and family preconceptions of palliative care and patient’s clinical profile influencing referral. The provider themes were conflict, making decisions and education needs. The systemic themes discovered were accessing services and resources and improving models of care.
Stocker et al., 2017 [45] United Kingdom	Longitudinal grounded theory qualitative study	14 professionals (of which 3 were specialist heart failure nurses), 17 patients with heart failure and 10 carers. Advanced heart failure palliative care	90%	To explore experiences of giving or receiving a prognosis and advanced palliative care planning for those with heart failure.	Lack of knowledge/notions about heart failure as a terminal illness in favour of a focus on day-to-day management and maintenance, despite obvious deterioration in disease stage and needs over time. Health professionals revealed frustration about the uncertainty of heart failure prognosis, leading to difficulties in planning care; need to deliver problem-based, individualized care; the lack of multidisciplinary advanced palliative care planning. Patients reported an absence of prognostic discussions with clinicians.
Wotton et al., 2005 [46] Australia	Qualitative descriptive study	17 nurses including registered nurses, clinical nurse consultants and clinical nurse or nurse managers. End-stage heart failure palliative care	80%	To describe the perception of registered nurses on factors influencing palliative care for patients with end-stage heart failure.	Potential barriers are correlated with the difficulty of really knowing the patient, the patient and family knowledge of the disease trajectory and relative awareness. Interdisciplinary team communications and clinical pathways are an obstacle to palliative care implementation. Another aspect is inexperience of correct treatment of symptoms.
Ziehm et al., 2016 [47] Germany	Qualitative study	23 healthcare professionals (nurses, physicians) with 4 hospital nurses, 3 outpatient care nurses, 4 heart failure nurses, and 1 palliative care nurse. Heart failure palliative care.	80%	To assess healthcare professionals’ attitudes regarding palliative care of chronic heart failure patients to identify barriers and facilitators for this patient group and thus to develop recommendations for the improvement of chronic heart failure patients’ access to palliative care in Germany.	Potential barriers are correlated with the unpredictable course of the disease; the acceptance of mortality by the patient and the fact that nurses and physicians do not educate them completely. Nursing staff would initiate palliative care earlier than physicians. Participants believe that it would be very important to have good communication and to implement education. They recognize the importance of palliative care for chronic heart failure.
Aiken et al., 2006 [28] Arizona	Randomized controlled study	240 patients of which 130 with chronic heart failure class IIIB or IV. Congestive heart failure home palliative care	70%	To assess the impact of the Phoenix program. A program which aims to reach patients receiving treatment from one of multiple managed care organizations.	Phoenix participants reported a sense of having greater information for self-management of illness. They showed a higher rate of having a living will or advanced directive. The physical functioning and general health improved. No significant effect on medical utilization emerged.
Bakitas et al., 2020 [29] USA	Randomized controlled study	415 patients: 208 IG, 207 CG. Palliative care for veterans with NYHA III–IV heart failure or American College of Cardiology stage C or D heart failure	85%	To determine the effect of an early palliative care telehealth intervention over 16 weeks on the quality of life, mood, global health, pain, and resource use of patients with advanced heart failure.	ENABLE CHF-PC, a nurse-led, early palliative care telehealth intervention did not demonstrate significant differences in quality of life or mood compared with usual care over 16 weeks. The secondary outcomes of pain intensity and pain interference demonstrated improvement, whereas global health and resource use were not different between the groups.
Bekelman et al., 2024 [30] Colorado, Washington	Single-blind two-group randomized clinical trial	IG 143 patients, CG: 147 patients. Home palliative care to veteran patients with chronic obstructive pulmonary disease or chronic heart failure or interstitial lung disease	77%	To evaluate the effect of a nurse and social worker palliative telecare team on the primary outcome of quality of life in adult outpatients with chronic obstructive pulmonary disease, chronic heart failure and interstitial lung disease.	The quality of life at six months after intervention was improved. The nurse and social worker telecare team also improved heart failure status.
Ng & Wong, 2018 [32] Hong Kong	Two-group randomized controlled trial	IG: 41 patients, CG: 43 patients. End-stage heart failure with a functional class of 3 to 4, not candidate for cardiac interventional therapy and who do not have a reversible precipitant	85%	To examine whether a home-based palliative heart failure program has effects on patient-related outcomes, including quality of life, symptom burden, satisfaction with care, and caregiver burden.	Significant improvement in the physical, psychological and existential domains, but not in the support domain of quality of life. Significant improvement in the dyspnoea, emotional function and mastery. Better improvement of depression and shortness of breath of symptom burden at 4 weeks. No difference in functional status, Higher satisfaction in intervention group. Significant decreasing burden at four weeks for caregiver.
Mirshani et al., 2024 [31] Iran	Pilot randomized controlled trial	50 patients: 22 IG and 23 CG. Home palliative care to patients with a diagnosis of New York Heart Association (NYHA) class II or III heart failure (HF) or American College of Cardiology (ACC) stage B or C HF.	85%	To determine the feasibility and acceptability of implementing an early telehealth palliative care intervention for heart failure patients.	This nurse-led, early telehealth palliative care intervention demonstrated evidence of feasibility, acceptability, and potential improvement on quality of life in patients with heart failure in Iran.
Rogers et al., 2017 [33] Not Specified	Randomized controlled study	150 patients: 75 IG and 75 CG. Chronic heart failure palliative care	62%	To assess the impact of an interdisciplinary palliative care intervention combined with usual heart failure management on heart failure-related and overall quality of life in patients with advanced heart failure.	An interdisciplinary palliative care intervention in the overall management of patients with advanced heart failure is beneficial.
Turrise et al., 2021 [53] North Carolina	Pilot pre- and post-test study	28 nurses providing direct care to people with heart failure Heart failure palliative and end-of-life care	83%	To determine the feasibility of an online, asynchronous module on the timing and content of palliative care conversations regarding nurses’ perceived knowledge and skill in having these conversations with people who have heart failure and their families.	Asynchronous education on the timing and content of palliative care conversations with people affected by heart failure improved nurse’s knowledge, perceived skill and comfort with having discussions.
Wong et al., 2016 [34] Hong Kong	Multi-site randomized controlled study	IG: 43 patients, CG: 41 patients. End-of-life home care palliative care with at least two of these criteria: CHF New York Heart Association (NYHA) stage III or IV, patient thought to be in the last year of life by clinicians, repeated hospital admissions (three within 1 year) with symptoms of heart failure and existence of physical/psychological symptoms despite optimal tolerated therapy	77%	To examine the effects of home-based transitional palliative care for patients with end-stage heart failure after hospital discharge.	Lower but not significant 4-weeks readmission rate in the intervention group. At 12 weeks, the readmission rate was significantly lower. The intervention group experienced significantly higher clinical improvements in depression and dyspnoea. Quality of life was associated with a significant improvement. The intervention group had significantly higher satisfaction with care than the control group.
Bharani et al., 2024 [48] United States	Descriptive study	2019: 110 IG and 100 CG patients. 2020: 110 IG and 170 CG patients. 2021: 97 IG and 93 CG patients.Advanced heart failure palliative care	55%	To evaluate if a specialty- trained palliative care nurse practitioner on the advanced heart failure team increases access to palliative care among people hospitalized with advanced heart failure earlier in their trajectory.	The number of palliative care consultations increased with the number of people identified earlier in their hospital and illness trajectory; the individuals seen by the embedded-based model were younger, more functional, more likely to have the capacity to designate a medical decision-maker, more likely to report life-prolonging goals of care, and more likely to discharge home, whereas those seen by referral-based model were older, more functionally impaired, fewer had capacity to designate a medical decision-maker, and they more frequently died in hospital.
Barret & Connaire., 2016 [49] Ireland	Cross-sectional descriptive design	76 nurses working in the critical care unit and cardiology ward. Heart failure palliative care	50%	To examine the knowledge and attitudes of cardiac nurses of a palliative care approach when caring for heart failure patients.	Palliative care needs of heart failure patients require education and training; this study identified specific educational needs in pain and symptom control and communication skills
Kim et al., 2020 [50] Seoul, Korea	Cross sectional descriptive design	102 nurses working in six general wards and three intensive critical units. Chronic heart failure tertiary hospital palliative care	63%	To examine palliative care knowledge, attitudes, confidence, and educational needs in nurses who care for patients with chronich heart failure, stroke, end-stage renal disease, and end-stage liver disease; explore the relationships between nurses’ palliative care knowledge, attitudes, confidence, and educational needs; and identify factors affecting nurses’ confidence in providing palliative care.	Nurses’ palliative care knowledge level was low and their attitude toward palliative care was moderate. Knowledge was significantly correlated with attitude. Previous training in hospice, palliative, and end-of-life care was a significant and modifiable factor that affected nurses’ confidence.
O’Hanlon et al., 2011 [43] United Kingdom	Cross-sectional self-report survey	142 British heart foundation specialist heart failure nurses. Heart failure palliative care	Cross-sectional tool: 88%Qualitative tool: 40%	To describe the current palliative care skills and knowledge of specialist heart failure nurses.	Nurses feel very strongly that it is their role to provide symptom control and psychological support. But they were less sure whether their role was to provide palliative care and bereavement support. Nurses identified two gaps in their training—first, communication skills, and second, a perceived lack of definition of the role of palliative care. Nurses felt that improving their communication skills would be very important and that they would benefit greatly from attending advanced communication skills training. The lack of definition of the role of palliative care was reflected in both a described need for symptom control skills for patients and confusion about the roles of palliative care, who was meant to be delivering and when palliative care should be initiated within the patient’s disease trajectory.
Pattenden et al., 2013 [51] United Kingdom	Prospective pragmatic nonrandomized controlled study	99 patients IG and 98 patients CG. Advanced congestive heart failure palliative care in primary setting	50%	To prove that a collaborative home-based palliative care service for patients with advanced chronich heart failure may increase the likelihood of death in place of choice and reduce inpatient admissions.	Using advanced care planning and recommended protocols has a positive impact on end-of-life care for patients with advanced chronich heart failure. The intervention was associated with reduced costs and increased benefits. Intervention group patients were more likely to die at home and less likely to die in hospital. Admission rates were lower in both groups. Unpredictable prognosis need not be a barrier to provision of end-of-life care services for patients with chronic heart failure.
Singh et al., 2021 [52] Australia and New Zeland	Survey	113 including 75 nurses, 32 physicians and 4 health professionals. Heart failure palliative care	33%	To determine the attitudes of cardiovascular healthcare professionals in Australia and New Zealand towards end-of-life care and its impact on specialist palliative care referral. Determine the association between end-of-life attitudes, the cardiovascular healthcare professionals’ self-reported delivery of supportive care and the healthcare professionals’ characteristics.	Some nurses agreed they experienced a sense of failure when they were not able to change the natural progression of heart failure or slow clinical worsening. Most nurses also prefer to refer patients to palliative care if they are classified in NYHA I-II. All agreed to refer a chronic heart failure patient who is experiencing a poor response to treatment and is in NYHA class IV.
Hjelmfors et al., 2014 [42] Sweden	Survey	114 heart failure nurses. Heart failure outpatients’ palliative care	Prevalence tool: 56% Qualitative tool: 30%	To describe heart failure nurses’ perspectives on, and daily practice regarding, the discussion of prognosis and end-of-life care with heart failure patients in outpatient care. To explore barriers, facilitators and related factors for discussing these issues.	A physician should have the main responsibility of discussing prognosis and end-of-life care, but also nurses could discuss these issues, especially if the patient initiates the discussion. Nurses perceive many barriers to discussions.

Legend: quality score—percentage of “yes” on total items of jbi critical appraisal checklist; IG—intervention group; CG—control group.

**Table 3:** Findings for each TDF domain.

Knowledge		Skills	
Patients and family		aQL 7.	The lack of master’s degrees in palliative care leads to a lack of palliative care nursing specialists to provide end-of-life care.
cQL 8.	Patient and their next of kin are not sufficiently informed by the doctor.	bQL 11.	Nurses are more able to talk to patients about death instead of doctors.
dQL. 1	Patients believe that by deactivating the ICD they will suddenly die.	fQL 11.	Nurses know that relapses are indicators that they are going into palliative care.
dQL 2.	Unrealistic patients’ expectation on diagnosis and trajectory obstacle communication about prognosis.	iQL 3.	There is the need to develop skill in dealing with end of life, understanding also when to start palliative care.
gQL 6.	Knowledge and information given to patients and family are important, as is talking about heart failure trajectory.	iQT 1.	Developing communication and bereavement skill also though training is important
Professionals		gQL 7.	Nurses perceived the importance of having the courage to talk about end-of-life care and giving hope to patients.
cQL 7.	Information about death is not always given by doctors.	jQL 4.	Identifying when access to palliative care is adequate is difficult.
hQL 4.	A gap for identifying who is responsible for providing palliative care exists.	jQL 5.	Palliative care involvement often happens too late.
jQL 6.	Cardiologists are often lacking in palliative care knowledge and palliative care professionals have a deficit in chronic heart failure management.	fQT 2.	A good relationship with the patient is important for communication.
jQL 7.	Professionals know that each heart failure patient is unique.	lQL 11.	The relationship with the patient has to be constructed starting from the beginning of the recovery.
jQL 8.	Patients have to know all treatment options and what palliative care is.	lQT 2.	The access to palliative care is dependent on heart failure, gravity and symptoms’ manifestation.
jQL 9.	Professionals believe palliative care is associated with cancer death and end-of-life.		
kQL 3.	Professionals know that heart failure can be managed but not cured.	Social/Professional Role and Identity	
mQL 1.	Chronic heart failure prevalence is increasing and palliative care needs to be expanded.	bQL 2.	Palliative care team involvement is important to offer a good death.
dQT 1.	Nurse education on death and dying is not always ensured.	bQL 3.	Teams’ discussions of cases and difficult death are fundamental to learning.
dQT 2.	Knowledge improves attitudes for dying patients.	cQL 10.	Being able to communicate, ask for advice and reflect on difficult situations is described as important.
lQL 1.	Nurses need to know palliative and chronic illness concepts in care.	cQL 11.	Communicat with colleagues is important and it could be painful or helpful in work
lQL 2.	Work as a team is a concept that nurses have to understand.	dQL 5.	Defining roles and duties is perceived as important.
lQL 3.	Physicians and nurses do not know how to delay symptoms.	eQT 1.	Interprofessional telecare intervention could improve health status.
		fQL 12.	Palliative care is becoming a much more understood as a nurse job.
Beliefs about capabilities		gQL 8.	Interprofessional teamwork is important, and communication training is needed.
aQL 8.	Communication between members of an interdisciplinary team is perceived as weak and fragmented but fundamental to providing palliative care.	jQT 1.	A supportive interprofessional palliative care program has a positive impact on cost and hospital admission.
bQL 4.	Communication between the interdisciplinary team, the patient and the family is important to achieving good death.	mQL 5.	Communication and cooperation in the team are important.
cQL 9.	Nurses understand the risk of forgetting social and existential needs when focusing on treatment.	jQL 13.	Having a multidisciplinary team with a coordinator in seen as beneficial.
fQT 2.	Nurses’ beliefs that discussing prognosis is a physician’s responsibility.	jQL 12.	Multiple professionals around the patient offering multiple ideas about the pathway can be an obstacle.
gQL 3.	Nurses hesitate to talk about end of life to patients if clinicians have not already done so.	lQL 9.	Poor communication in numerous teams of professionals detracted from effectively planning of palliative care.
iQL 1.	Nurses do not feel comfortable with death.	lQL 10	Nurses in cardiac wards desire greater palliative care team consultation.
iQL 2.	Understanding when saying the right thing is difficult.		
jQL 14.	Nurses and health care professionals do not feel comfortable discussing palliative care with a patient.	Optimism	
jQL 15.	Nurses recognise the palliative care need but do not want to refer without a cardiologist.	Patients and caregivers	
fQT 1.	Nurses perceive a lack of education in discussing prognosis and do not initiate discussion unless the patient initiates first.	bQL 6.	Patient false hope is an obstacle to good death.
		bQL 6.	Families are described as reluctant to accept that further intervention is unrealistic.
Beliefs about consequences		bQL 7.	To be on the transplant list is considered as being saved.
bQT 1.	Nurse practitioner involvement has a positive impact on palliative care access and patient’s goals.	dQL 3.	Patients are optimistic because they do not have cancer.
fQL 9.	Talking to people about the end of life is difficult.	eQL 1.	Patients do not recognise a chronic condition as a life-limiting illness.
fQL 10.	With discussion about end-of-life, nurses do not want to frighten people or take away their hope.	mQL 4.	Patients do not want to end active therapy because they do not accept their mortality.
eQL 4.	Health professional (doctors and nurses) education allows the integration of palliative care.	Professionals	
eQL 5.	Health professional education favors terminal sign recognition.	eQL 2.	Clinicians’ knowledge on how to recognize patients in the last years of life allows advanced care planning with family involvement.
jQL 2.	Patients and family are resistant and reluctant to palliative care, perceived as giving up.	lQL 4.	Professionals believe that patients deny or were unaware of the gravity of the illness.
jQL 3.	Palliative care is perceived as giving up for patients, family and professionals.	lQL 5.	Younger nurses do not comprehend mortality because they think they are immortal.
mQT 1.	Specific educational intervention is beneficial in improving communication in heart failure palliative care.		
		Intentions	
Goals		aQL 9.	The team spirit for effective team working to provide palliative care is lacking.
bQL 1	It is important that patients and relatives accept death to talk about good death.	gQL 4.	Discussing prognosis as part of clinical practice and involving the palliative care team is important.
cQL 5.	Uncertainty characterized the course of illnesses and how to manage patient care.	gQL 4.	Patients do not always want to talk about prognosis.
cQL 6.	It is difficult to understand if focusing on symptoms and medical treatment could cloud social and existential needs.	jQL 1.	Some people are happy to discuss prognosis while others do not want to.
fQL 5.	Clinicians’ priorities are not aligned with nurses’ ones about goals of treatment.	kQL 2.	Clinicians desire to talk about prognosis at the time of the diagnosis, but it is not always possible.
fQL 6.	Clinicians prefer to fix things and sometimes it is not possible.	lQL 14.	Physicians perceived transition to palliative care as a failure.
fQL 7.	Illness trajectory uncertainty obstructs conversation.		
fQL 8.	Patients do not want involvement in palliative care if nurses believe that they will live more than a short amount of time.	Environmental context and resources	
gQL 1.	Heart failure clinic is not contemplated as a place to talk about end-of-life	aQL 5.	The lack of specialist medical professionals in the field of palliative care is perceived as challenging when providing end-of-life care for those with heart failure.
gQL 2.	When heart failure treatment is still possible patients are not considered in a terminal phase.	aQL 6.	Palliative care centers that provide palliative care and meet physical, spiritual and psychological needs have not been considered by the Iranian health system.
iQT 2.	There is confusion in understanding the definition of palliative care and the correct time to take the step.	eQL 3.	Financial constraints and time prevent nurses from talking and educating people.
jQL 10.	Cardiologists fixate on heart failure mechanisms, not on well-being.	hQL 1.	Government and insurance companies do not support palliative care companies.
jQL 11.	Professionals want to be honest with the patient about goals.	hQL 2.	Palliative care is dependent on benefactors and charities for financial support.
mQL 3.	Nurses and doctors perceive the need to initiate palliative care at different moments.	mQL 6.	Professional education in palliative care is a huge need.
fQT 3.	Unpredictable disease trajectory is perceived as a barrier.	jQL 16.	Palliative care services are limited, and professionals experience difficulties in involving and connecting with them.
lQL 7.	Patients are not aware of having a chronic long-term progressive illness.	lQL 12.	The time to construct a relationship with the patient is often limited
lQL 8.	Physicians focus on how to cure a particular stage and life-sustaining treatment, without thinking about death.	lQL 13.	Limited resources limit palliative care and the development of a personalized plan of care.
mQL 2.	It is difficult to understand when to start palliative care due to the unpredictable course of the disease.	cQL 4.	The homecare setting is viewed as easier in letting people be themselves.
		dQL 4.	Conversations between the cardiologist and patients on system, environment and time obstacles.
Social Influences		fQL 4.	Involvement is often only at the end of life, avoiding end-of-life conversations.
aQL 3.	No-resuscitation law has not yet been approved in some countries, so the patient could receive CPR when having a no resuscitation order.		
aQL 4.	A lack of support from the health system to provide palliative care services for those with heart failure patients at the end of their life is perceived.	Emotion	
hQL. 3	A gap for identifying who is responsible for providing palliative care exists.	aQL 1.	Nurses feel that taking care of end-of-life heart failure patients has made them nervous and constantly anxious.
lQL 6.	Western society does not want to introduce, speak or plan about death.	aQL 2.	Nurses feel very tired and have headaches and dizziness; they feel like they are drowning in the suffering of these patients.
		bQL. 8	Family denies and does not want to know when they can see that the patient is getting worse.
Behavioral regulation		bQL. 9	Nurses feel angry and frustrated when they have to resuscitate people who they identify as not appropriate for resuscitation.
aQT 1.	Nurse case manager involvement with a leadership role; disease and symptom management has a positive impact on frequency and severity of symptoms.	cQL 1.	Nurses perceive it positively when patients rely on them communicating their feelings.
aQT 2.	Nurse case manager involvement with a leadership role, disease and symptom management has no different impact on physical and mental functioning.	cQL 3.	Nurses share with the workgroup a feeling of tiredness and being worn-out.
cQT 1.	Nurse-led psychoeducational interventions may be beneficial for quality of life.	fQL 1.	Patients and family are welcome to engage in conversation about end of life.
gQT 1.	Post-discharge nurse case managers performing home visits and telephone calls could be beneficial for quality of life, patient’s symptoms and satisfaction.	fQL 2.	Nurses carry the heavy knowledge that one patient will not go home.
hQT 1.	Virtual nurse programs could improve quality of life.	fQL 3.	Nurses describe frustration when nobody makes the decision about palliative care.
hQT 2.	Nurse virtual programs produce no difference in mood status and emergency department visits are reduced.	cQL 2.	An inadequate feeling emerges when it is difficult to talk about death in a homecare setting.
kQT 1.	An interdisciplinary palliative intervention coordinated by nurse practitioners could improve quality of life and psychological symptoms.	kQL 1.	Heart failure for the patient is a scary term, which creates denial or upset feelings.
nQT 1.	A nurse case manager’s palliative care program could have a positive impact on hospital readmission, patients satisfaction and some symptoms.	lQT 1.	A sense of failure was expressed because it is not possible to change the natural progression of heart failure.

Legend: aQT [28], aQL [35], bQT [48], cQT [29], dQT [49], eQT [30], bQL [36], cQL [37], dQL [38], eQL [39], fQL [41], gQL [42], fQT [42], hQT [31], gQT [32], iQL [43], iQT [43], jQT [51], kQT [33], jQL [44], kQL [45], lQT [52], lQL [46], mQT [53], mQL [47], nQT [34].

**Table 4:** Quantitative studies’ interventions and outcomes explored.

	Phsycological Aspect	Quality of life\Health Status	Symtomp Severity	Physical Function	Palliative Care Access	Patient Satisfiction	Access Emergency Department	Cost of Care	Knowledge and Skills in Palliative Care Conversation
Aiken et al., 2006 [28]	⮽		☑	⮽					
	Registered nurse case managers delivered disease and symptom management, patient and caregiver education on disease management and social and psychological support, and assumed a leadership role in coordinating services								
Bharani et al., 2024 [48]					☑				
	A palliative care nurse practitioner was introduced into the advanced heart failure care								
Bakitas et al., 2020 [29]		☑ After 16 weeks of intervention							
	Nurse-led predominantly telephone-based psychoeducational intervention								
Bekelman et al., 2014 [30]		☑							
	Team intervention based on the effective collaborative care model. A registered nurse addressed symptoms and a social worker provided structured counseling through six phone calls planned twice a month.								
Ng & Wong, 2018 [32]		☑	☑ After 4 weeks, no difference at 12 weeks			☑			
	Post-discharge home visits and telephone calls delivered by palliative care nurse case managers provide physical and psychological symptoms’ assessments and management, social support, spiritual and existential aspects of care, and discussion of treatment preference and end of life issues								
Mirshahi et al., 2024 [31]	⮽	☑					⮽		
	A nurse interventionist conducted a comprehensive six-week virtual program with a weekly webinar and a six-chapter booklet entitled “palliative care in patients with heart failure”								
Pattenden et l., 2013 [51]							☑	☑	
	Marie Curie Cancer Care nurses and Marie Curie Cancer Care healthcare assistants working together alongside cardiologists, “care of the elderly” consultants, district nurses and general practitioners								
Rogers et al., 2017 [33]	☑	☑							
	A certified palliative care nurse practitioner coordinated the management of physical symptoms, psychological and spiritual concerns, and advanced care planning in collaboration with a hospice and palliative medicine board-certified physician.								
Turrise et al., 2021 [53]									☑
	Asynchronous educational intervention regarding communication on palliative and end-of-life heart failure care for nurses								
Wong et al., 2016 [34]	☑		☑			☑	☑		
	Nurse case managers delivered a palliative program service centered on case management, discussion of end-of-life issues, multidisciplinary approach, staff development for communication, discussion of treatment preferences, an integrated model of the case								

☑ Improved, ⮽ no difference.

**Table 5:** Summary of findings.

Systematic review title: Nursing attitudes and perceptions regarding heart failure palliative care: a mixed method systematic review Population: heart failure palliative care Phenomena of interest: nursing attitudes, experiences and perceptions of heart failure palliative care Context: Heart failure is correlated with important symptom burden. Heart failure is a noncommunicable disease in the treatment of which palliative care can be implemented.					
**Synthesized Finding in TDF Domain**		**Type of Research**	**Dependability**	**Credibility**	**ConQual Score**
Knowledge	Knowledge improves attitudes but a theoretical gap exists between professionals and patients in symptom management, heart failure trajectory and responsibility.	Qualitative and quantitative	Downgrade 1 level	Unchanged	Moderate
Skills	Skills such as communication, relationship, and correct time to start palliative care are difficult, and nurses are more well-equipped than clinicians.	Qualitative and quantitative	Downgrade 1 level	Downgrade 1 level	Low
Social/Professional Role and Identity	Interprofessional collaboration with a definition of duties is fundamental. Multiple ideas and poor communication could become an obstacle.	Qualitative and quantitative	Downgrade 1 level	Downgrade 1 level	Low
Beliefs about Capabilities	Developing communication abilities is important. Nurses are not comfortable discussing prognosis if they do not perceive it to be effective.	Qualitative and quantitative	Downgrade 1 level	Downgrade 1 level	Low
Optimism	False hope, limited awareness and lack of acceptance with unrealistic feelings are typical of patients and families.	Qualitative	Downgrade 1 level	Unchanged	Moderate
Beliefs about Consequences	Patients and families consider palliative care as giving up. Education is important for palliative care integration.	Qualitative and quantitative	Downgrade 1 level	Downgrade 1 level	Low
Intentions	Prognosis is a difficult topic to start if considered as important.	Qualitative	Downgrade 1 level	Downgrade 1 level	Low
Goals	Heart failure represents an unpredictable obstacle in the palliative care setting and forming agreements between professionals.	Qualitative and quantitative	Downgrade 1 level	Unchanged	Moderate
Environmental Context and Resources	Limited resources, financial constraints and little time to construct relationships limit care.	Qualitative	Downgrade 1 level	Unchanged	Moderate
Social Influences	Lack of health system support may be an obstacle.	Qualitative	Downgrade 1 level	Downgrade 2 level	Very Low
Emotion	Frustration, tiredness and nervousness emerged when having to deal with suffering with no clear pathway. Positive feelings when communication exists.	Qualitative	Downgrade1 level	Downgrade 1 level	Moderate
Behavioural regulation	Nurses’ coordination of care and educational intervention in heart failure palliative care and symptom management could improve patients’ outcomes.	Quantitative	Downgrade 1 level	Downgrade 1 level	Low

## Data Availability

The authors confirm that the data supporting the findings of this study are available within the article.

## Abbreviations

The following abbreviations are used in this manuscript:

ESC	European Society of Cardiology
LVAD	Left ventricular assistance device
JBI	Joanna Briggs Institute
PRISMA	Preferred Reported Items for Systematic review and Meta-analysis
QL	Qualitative
QT	Quantitative
TDF	Theoretical domains framework
WHO	World Health Organization
